# Reported Incidence of Infections Caused by Pathogens Transmitted Commonly Through Food: Impact of Increased Use of Culture-Independent Diagnostic Tests — Foodborne Diseases Active Surveillance Network, 1996–2023

**DOI:** 10.15585/mmwr.mm7326a1

**Published:** 2024-07-04

**Authors:** Hazel J. Shah, Rachel H. Jervis, Katie Wymore, Tamara Rissman, Bethany LaClair, Michelle M. Boyle, Kirk Smith, Sarah Lathrop, Suzanne McGuire, Rosalie Trevejo, Marcy McMillian, Stic Harris, Joanna Zablotsky Kufel, Kennedy Houck, Carey E. Lau, Carey J. Devine, Dave Boxrud, Daniel L. Weller

**Affiliations:** ^1^Division of Foodborne, Waterborne, and Environmental Diseases, National Center for Emerging and Zoonotic Infectious Diseases, CDC; ^2^Colorado Department of Public Health and Environment; ^3^California Emerging Infections Program, Oakland, California; ^4^Connecticut Emerging Infections Program, Yale School of Public Health, New Haven, Connecticut; ^5^Georgia Department of Public Health; ^6^Maryland Department of Health; ^7^Minnesota Department of Health; ^8^University of New Mexico, Albuquerque, New Mexico; ^9^New York State Department of Health; ^10^Oregon Health Authority; ^11^Tennessee Department of Health; ^12^Center for Food Safety and Applied Nutrition, Food and Drug Administration, College Park, Maryland; ^13^Food Safety and Inspection Service, U.S. Department of Agriculture, Washington, DC.

**Summary**

**What is already known about this topic?**

Increased use of culture-independent diagnostic tests (CIDTs) affects observed trends in foodborne infection incidence.

**What is added by this report?**

During 2023, the incidence of eight domestically acquired infections transmitted commonly through food either increased or remained stable compared with 2016–2018, the baseline used to track progress toward disease reduction goals. Incidence of CIDT-diagnosed infection also increased during 2023.

**What are the implications for public health practice?**

CIDTs allow for diagnosis of infections that previously would have been undetected; recent increases in incidence appear to be driven by increased CIDT use. Continued surveillance is needed to monitor the impact of changing diagnostic practices on disease trends. Targeted prevention efforts are needed to reduce disease incidence.

## Abstract

Reducing foodborne disease incidence is a public health priority. This report summarizes preliminary 2023 Foodborne Diseases Active Surveillance Network (FoodNet) data and highlights efforts to increase the representativeness of FoodNet. During 2023, incidences of domestically acquired campylobacteriosis, Shiga toxin-producing *Escherichia coli* infection, yersiniosis, vibriosis, and cyclosporiasis increased, whereas those of listeriosis, salmonellosis, and shigellosis remained stable compared with incidences during 2016–2018, the baseline used for tracking progress towards federal disease reduction goals. During 2023, the incidence and percentage of infections diagnosed by culture-independent diagnostic tests (CIDTs) reported to FoodNet continued to increase, and the percentage of cases that yielded an isolate decreased, affecting observed trends in incidence. Because CIDTs allow for diagnosis of infections that previously would have gone undetected, lack of progress toward disease reduction goals might reflect changing diagnostic practices rather than an actual increase in incidence. Continued surveillance is needed to monitor the impact of changing diagnostic practices on disease trends, and targeted prevention efforts are needed to meet disease reduction goals. During 2023, FoodNet expanded its catchment area for the first time since 2004. This expansion improved the representativeness of the FoodNet catchment area, the ability of FoodNet to monitor trends in disease incidence, and the generalizability of FoodNet data.

## Introduction

Reducing the incidence of foodborne and enteric diseases is a public health priority. The Healthy People 2030 (HP2030) initiative established disease reduction goals for *Campylobacter, Listeria*, *Salmonella,* and Shiga toxin-producing *Escherichia coli* (STEC) infections (*1*). To evaluate progress toward HP2030 goals, CDC’s Foodborne Diseases Active Surveillance Network (FoodNet) monitors infections caused by eight pathogens transmitted commonly through food. This report summarizes preliminary 2023 surveillance data and describes changes in incidence compared with average annual incidence during 2016–2018, the reference period used by HP2030 (*1*).

## Methods

### Data Source

FoodNet conducts active, population-based surveillance for laboratory-diagnosed *Campylobacter, Cyclospora, Listeria, Salmonella,* *Shigella,* STEC, *Vibrio,* and *Yersinia* infections and pediatric hemolytic uremic syndrome (HUS) at 10 U.S. sites;* HUS is monitored because it can be a complication of STEC infection. FoodNet’s catchment area expanded during 2023 to include all of Colorado, and now represents 16% of the U.S. population (53.6 million persons); in 2023, the historic catchment area represented 15% of the U.S. population (51.0 million persons). Compared with the historic catchment area, the expansion increased representation for specific populations, including Hispanic or Latino ([Hispanic]; 8% increase), American Indian or Alaska Native (AI/AN; 8% increase), and Native Hawaiian or Pacific Islander (NH/PI; 6% increase) persons (FoodNet collects race and ethnicity as separate variables) as well as persons living in rural counties (10% increase).

### Laboratory Testing and Data Collection

Bacterial infections were diagnosed by culture or culture-independent diagnostic tests (CIDTs). Cyclosporiasis was diagnosed by polymerase chain reaction or microscopy. Pediatric HUS surveillance is conducted through a network of nephrologists and infection preventionists and by hospital discharge data review.^†^ This report includes 2022 data on pediatric HUS cases, the most recent year for which data are available. This activity was reviewed by CDC, deemed not research, and conducted in accordance with applicable federal law and CDC policy.^§^

### Statistical Methods

Bayesian negative binomial models were implemented to estimate changes in incidence in the historic catchment area during 2023 compared with average annual incidence during 2016–2018 (overall, and for domestically acquired infections), using R statistical software (version 2.14.0; R Foundation).^¶^^,^** Incidence in 2023 was considered substantially different^††^ from that during 2016–2018 if the 95% credible interval (CrI) for the incidence rate ratio (IRR) did not include 1.0. Cross-tabulations by demographic and other characteristics were also performed.^§§^

## Results

### Incidence in 2023 Compared with Average Annual Incidence During 2016–2018

During 2023, FoodNet identified 29,607 infections, 7,234 hospitalizations, and 177 deaths overall (including domestically acquired and travel-associated infections) in the historic catchment area (Table 1) (Table 2), compared with 31,492 infections, 7,588 hospitalizations, and 184 deaths in the expanded catchment area^¶¶^ (Supplementary Table; https://stacks.cdc.gov/view/cdc/157822). In both the historic and expanded catchment areas, 15% of cases were associated with international travel. Overall, and for domestically acquired infections only, incidence of campylobacteriosis was highest, followed by salmonellosis and STEC infection. In the historic catchment area during 2023, incidences of domestically acquired campylobacteriosis, cyclosporiasis, STEC infection, vibriosis, and yersiniosis increased compared with those during 2016–2018, whereas listeriosis, salmonellosis, and shigellosis incidences remained stable.

**TABLE 1:** Demographic characteristics of persons with laboratory-diagnosed bacterial and parasitic infections during 2023* in the historic^†^ and expanded^§^ catchments compared with the overall population of each catchment — Foodborne Diseases Active Surveillance Network, United States, 2023

Characteristic	Historic catchment, no. (%)		Expanded catchment, no. (%)		% Increase^¶^	
	Cases	Catchment	Cases	Catchment	Cases	Catchment
**Total**	**29,607**	**51,000,988**	**31,492**	**53,588,559**	**6.4**	**5.1**
**Age group, yrs**						
≤4	3,534 (11.9)	2,776,555 (5.4)	3,733 (11.9)	2,914,934 (5.4)	5.6	5.0
5–17	2,611 (8.8)	8,123,969 (15.9)	2,780 (8.8)	8,533,731 (15.9)	6.5	5.0
18–59	14,806 (50.0)	28,111,004 (55.1)	15,738 (50.0)	29,538,011 (55.1)	6.3	5.1
≥60	8,656 (29.2)	11,989,460 (23.5)	9,241 (29.3)	12,601,883 (23.5)	6.8	5.1
Not reported	0 (—)**	—**	0 (—)**	—**	—**	—**
**Sex**						
Female	14,577 (49.2)	25,810,919 (50.6)	15,565 (49.4)	27,076,136 (50.5)	6.8	4.9
Male	14,966 (50.6)	25,190,069 (49.4)	15,863 (50.4)	26,512,423 (49.5)	6.0	5.2
Not reported	64 (0.2)	—**	64 (0.2)	—**	—**	—**
**Ethnicity^††^**						
Hispanic or Latino	4,041 (13.6)	6,786,543 (13.3)	4,428 (14.1)	7,348,445 (13.7)	9.6	8.3
Not Hispanic or Latino	21,575 (72.9)	44,214,445 (86.7)	23,011 (73.1)	46,240,114 (86.3)	6.7	4.6
Not reported	3,991 (13.5)	—**	4,053 (12.9)	—**	1.6	—**
**Race**						
AI/AN	254 (0.9)	676,635 (1.3)	263 (0.8)	728,099 (1.4)	3.5	7.6
Asian	1,562 (5.3)	3,263,553 (6.4)	1,574 (5.0)	3,317,521 (6.2)	0.8	1.7
Black or African American	3,430 (11.6)	8,743,160 (17.1)	3,471 (11.0)	8,825,882 (16.5)	1.2	0.9
NH/PI	54 (0.2)	100,062 (0.2)	60 (0.2)	106,048 (0.2)	11.1	6.0
White	19,599 (66.2)	36,674,840 (71.9)	21,147 (67.2)	38,980,732 (72.7)	7.9	6.3
Other	1,895 (6.4)	—**	2,024 (6.4)	—**	6.8	—**
Multiple races	413 (1.4)	1,542,738 (3.0)	452 (1.4)	1,630,277 (3.0)	9.4	5.7
Not reported	2,400 (8.1)	—**	2,501 (7.9)	—**	4.2	—**
**Urbanicity^§§^**						
Urban	24,565 (83.0)	43,609,552 (85.5)	25,740 (81.7)	45,481,094 (84.9)	4.8	4.3
Rural	5,041 (17.0)	7,391,436 (14.5)	5,751 (18.3)	8,107,465 (15.1)	14.1	9.7

**Abbreviations:** AI/AN = American Indian or Alaska Native; FoodNet = Foodborne Diseases Active Surveillance Network; NH/PI = Native Hawaiian or Pacific Islander; RUCC = rural-urban continuum code.

* Case data for 2023 are preliminary.

^†^ When FoodNet was founded in 1996, the catchment included Minnesota and Oregon and counties in California (two), Connecticut (two), and Georgia (eight). The catchment expanded consistently during 1996–2004 and remained stable during 2004–2022. The historic catchment includes sites under surveillance since 2004, including Connecticut, Georgia, Maryland, Minnesota, New Mexico, Oregon, and Tennessee, and counties in California (three), Colorado (seven), and New York (34).

^§^ In 2023, the remaining 57 Colorado counties were enrolled in the FoodNet catchment. The expanded catchment includes those sites that were part of the historic catchment and the remaining 57 Colorado counties. Because yersiniosis is not a notifiable disease in Colorado, FoodNet only collected data on yersiniosis cases in the seven Colorado counties included in the historic catchment. Therefore, yersiniosis data for the historic and expanded catchments will be the same.

^¶^ Percent increase among persons with the given demographic characteristic in the expanded catchment compared with the historic catchment using the equation (No. in expanded – No. in historic) / (No. in historic) × 100.

** Dashes indicate that the given data point was unknown, not reported, or otherwise missing from the FoodNet data. The U.S. Census Bureau data used to describe catchment characteristics do not include a comparable “not reported” category for any of the characteristics of interest.

^††^ FoodNet's data collection mechanism includes separate questions about ethnic and racial identity. As a result, persons could identify as any combination of ethnicity and race.

**^§§^** Urbanicity was determined using RUCC (https://www.ers.usda.gov/data-products/rural-urban-continuum-codes/) for the county of residence. During 2020, U.S. Census Bureau data were generated for Connecticut planning regions instead of Connecticut counties; as a result, the 2023 RUCC estimates were calculated for Connecticut planning regions but not Connecticut counties. Because FoodNet collected county of residence (as opposed to planning region of residence) for Connecticut cases, the catchment population and all cases from all sites except Connecticut were stratified into urban and rural using 2023 RUCC data. Connecticut cases were classified as urban or rural using 2013 RUCC data; 2013 is the most recent year for which RUCC data are available for Connecticut counties.

**TABLE 2:** Number of laboratory-diagnosed infections, hospitalizations, deaths, outbreak-associated cases, and crude incidence in the historic* catchment area during 2023^†^ compared with 2016–2018 average annual incidence and Healthy People 2030 incidence targets,^§^ by pathogen overall and for domestically acquired infections only — Foodborne Diseases Active Surveillance Network, United States, 2016–2018 and 2023

Pathogen	Infections, no.^¶^	No. (%)			Crude average incidence,^¶¶ ^2016–2018	2023 incidence		IRR (95% CrI)^†††^	HP2030 incidence target^§§§^
		Hospitalizations**	Deaths^††^	Outbreak-associated^§§^		Crude***	Estimated (95% CrI)^†††^		
**All cases (including international travel–associated)^¶¶¶^**									
**Bacteria**									
*Campylobacter*	11,926	2,482 (20.8)	49 (0.4)	30 (0.3)	18.2	23.4	21.52 (20.34–22.75)	1.19 (1.11–1.26)	NA
Salmonella****	8,454	2,456 (29.1)	55 (0.7)	644 (7.6)	16.9	16.6	15.79 (14.89–16.75)	0.97 (0.91–1.04)	NA
*S. *Enteritidis	1,597	460 (28.8)	12 (0.8)	178 (11.1)	2.6	3.1	2.72 (2.47–3.00)	1.04 (0.93–1.16)	NA
*S. *Newport	566	179 (31.6)	0 (**—)^††††^**	23 (4.1)	1.6	1.1	1.26 (1.02–1.64)	0.80 (0.62–1.07)	NA
*S. *Typhimurium	541	154 (28.5)	1 (0.2)	77 (14.2)	1.4	1.1	1.21 (1.09–1.35)	0.84 (0.75–0.94)	NA
*S. *Javiana	324	114 (35.2)	4 (1.2)	10 (3.1)	1.2	0.6	0.73 (0.58–0.97)	0.59 (0.44–0.81)	NA
*S. *I 4,[5],12:i:-	279	79 (28.3)	3 (1.1)	36 (12.9)	0.9	0.5	0.56 (0.47–0.68)	0.65 (0.54–0.81)	NA
Other serotypes	2,850	920 (32.3)	22 (0.8)	277 (9.7)	5.9	5.5	5.43 (5.10–5.79)	0.92 (0.85–0.99)	NA
Not serotyped	2,297	550 (23.9)	13 (0.6)	43 (1.9)	2.6	4.6	4.56 (4.97–5.29)	1.74 (1.44–2.08)	NA
STEC^§§§§^	3,351	685 (20.4)	14 (0.4)	108 (3.2)	4.7	6.6	6.29 (5.62–7.07)	1.33 (1.16–1.52)	NA
non-O157**^¶¶¶¶^**	1,112	183 (16.5)	3 (0.3)	42 (3.8)	2.1	2.2	2.11 (1.83–2.46)	1.03 (0.87–1.23)	NA
O157	298	114 (38.3)	3 (1.0)	51 (17.1)	0.8	0.6	0.61 (0.53–0.69)	0.72 (0.62–0.82)	NA
Not serogrouped	1,941	388 (20.0)	8 (0.4)	15 (0.8)	2.1	3.8	4.03 (3.11–5.60)	1.93 (1.42–2.83)	NA
*Shigella*	3,186	969 (30.4)	9 (0.3)	124 (3.9)	4.8	6.2	5.44 (4.49–6.69)	1.13 (0.91–1.40)	NA
*Yersinia*	1,437	325 (22.6)	9 (0.6)	0 (**—)^††††^**	0.8	2.8	2.59 (2.28–2.97)	3.43 (2.94–4.06)	NA
*Vibrio*	567	118 (20.8)	5 (0.9)	10 (1.8)	0.7	1.1	1.15 (1.01–1.31)	1.69 (1.47–1.94)	NA
*Listeria*	163	159 (97.5)	36 (22.1)	5 (3.1)	0.3	0.3	0.30 (0.26–0.34)	1.13 (0.98–1.31)	NA
**Parasite**									
*Cyclospora*	523	40 (7.6)	0 (**—)^††††^**	40 (7.6)	0.3	1.0	1.56 (1.00–2.97)	4.75 (2.76–9.50)	NA
**Total**	**29,607**	**7,234 (24.4)**	**177 (0.6)**	**961(3.2)**	**—^††††^**	**—^††††^**	**—^††††^**	**—^††††^**	**—^††††^**
Domestically acquired cases only*****									
**Bacteria**									
*Campylobacter*	10,516	2,368 (22.5)	48 (0.5)	30 (0.3)	15.8	20.6	19.32 (18.30–20.41)	1.22 (1.15–1.30)	10.9
*Salmonella*	7,237	2,202 (30.4)	53 (0.7)	621 (8.6)	14.8	14.2	13.92 (13.10–14.81)	0.94 (0.87–1.01)	11.5
*S. *Enteritidis	1,238	398 (32.1)	10 (0.8)	167 (13.5)	2.1	2.4	2.13 (1.92–2.38)	1.01 (0.89–1.14)	NA
*S. *Newport	523	170 (32.5)	0 (**—)^††††^**	23 (4.4)	1.5	1.0	1.17 (0.94–1.55)	0.77 (0.58–1.06)	NA
*S. *Typhimurium	505	150 (29.7)	1 (0.2)	76 (15.0)	1.4	1.0	1.15 (1.03–1.29)	0.84 (0.74–0.95)	NA
*S. *Javiana	303	108 (35.6)	4 (1.3)	4 (1.3)	1.2	0.6	0.70 (0.55–0.95)	0.58 (0.43–0.81)	NA
*S.* I 4,[5],12:i:-	240	72 (30.0)	3 (1.3)	36 (15.0)	0.8	0.5	0.51 (0.42–0.62)	0.64 (0.52–0.80)	NA
Other serotypes	2,436	789 (32.4)	22 (0.9)	273 (11.2)	4.8	4.7	4.80 (4.49–5.15)	0.90 (0.83–0.98)	NA
Not serotyped	1,992	515 (25.9)	13 (0.7)	42 (2.1)	2.4	4.0	4.04 (3.51–4.71)	1.67 (1.38–2.01)	NA
STEC	2,703	617 (22.8)	14 (0.5)	103 (3.8)	4.1	5.3	5.15 (4.63–5.75)	1.25 (1.10–1.42)	3.7
non-O157^†††††^	882	160 (18.1)	3 (0.3)	39 (4.4)	1.7	1.7	1.70 (1.47–1.98)	1.00 (0.85–1.19)	NA
O157	276	111 (40.2)	3 (1.1)	50 (18.1)	0.8	0.5	0.57 (0.50–0.65)	0.71 (0.61–0.81)	NA
Not serogrouped	1,545	346 (22.4)	8 (0.5)	14 (0.9)	1.8	3.0	3.19 (2.52–4.26)	1.79 (1.36–2.48)	NA
*Shigella*	2,370	886 (37.4)	8 (0.3)	123 (5.2)	4.1	4.6	4.21 (3.42–5.26)	1.02 (0.81–1.29)	NA
*Yersinia*	1,376	319 (23.2)	9 (0.7)	0 (**—**)**^††††^**	0.7	2.7	2.49 (2.18–2.85)	3.47 (2.97–4.08)	NA
*Vibrio*	501	108 (21.6)	5 (1.0)	10 (2.0)	0.6	1.0	1.02 (0.90–1.16)	1.64 (1.43–1.90)	NA
*Listeria* ^§§§§§^	160	156 (97.5)	36 (22.5)	5 (3.1)	0.26	0.31	0.29 (0.26–0.34)	1.13 (0.98–1.32)	0.22
**Parasite**									
*Cyclospora*	367	34 (9.3)	0 (**—**)**^††††^**	39 (10.6)	0.3	0.7	1.25 (0.73–2.87)	5.06 (2.65–12.43)	NA
**Total**	**25,230**	**6,690 (26.5)**	**173 (0.7)**	**931 (3.7)**	**—^††††^**	**—^††††^**	**—^††††^**	**—^††††^**	**—^††††^**

**Abbreviations:** CIDT = culture-independent diagnostic test; CrI = credible interval; FoodNet = Foodborne Diseases Active Surveillance Network; HP2030 = Healthy People 2030; IRR = incidence rate ratio; NA = not applicable; STEC = Shiga toxin-producing *Escherichia coli*.

* When FoodNet was founded in 1996, the catchment included Minnesota and Oregon and counties in California (two) Connecticut (two), and Georgia (eight). The catchment expanded consistently during 1996–2004; it remained stable during 2004–2022. The historic catchment includes sites under surveillance since 2004, including Connecticut, Georgia, Maryland, Minnesota, New Mexico, Oregon, and Tennessee, and counties in California (three), Colorado (seven), and New York (34). To facilitate comparability with past FoodNet reports, data for the historic catchment are presented in-text.

^†^ Case data for 2023 are preliminary.

^§^ HP2030 is a 10-year plan for addressing critical public health priorities and challenges. U.S. Department of Health and Human Services releases priority objectives as part of HP2030, including incidence targets for *Campylobacter*, *Salmonella*, STEC, and *Listeria* infections, to be met by 2030. https://health.gov/healthypeople/objectives-and-data/browse-objectives/foodborne-illness

^¶^ Bacterial infections were diagnosed using culture or culture-independent diagnostic tests. *Cyclospora* infections were diagnosed using microscopy or polymerase chain reaction.

** Admission to an inpatient unit or an observation stay of >24 hours ≤7 days before or after specimen collection or determined to be related to the infection if beyond this time frame. The average percentage of infections resulting in hospitalizations during 2016–2018, by pathogen, were *Campylobacter* (20%), *Salmonella* (27%), STEC (22%), *Shigella* (24%), *Yersinia* (26%), *Vibrio* (30%), *Listeria* (96%), *Cyclospora* (6%), and overall (24%). Infections with unknown hospitalization status (6% of infections during 2023 and 4% during 2016–2018) were included in the denominator only.

**^††^** Attributed to infection when death occurred during hospitalization or ≤7 days after specimen collection from nonhospitalized patients. The average percentage of infections resulting in death during 2016–2018 were, by pathogen, *Campylobacter* (0.4%), *Salmonella* (0.4%), STEC (0.4%), S*higella* (0.1%), *Yersinia* (1.2%), *Vibrio* (2.1%), *Listeria* (18.6%), *Cyclospora* (0.2%), and overall (0.5%). Infections with unknown death status (8% of infections during 2023 and 3% during 2016–2018) were included in the denominator only.

^§§^ Generally defined as two or more cases of similar illness associated with a common exposure; some sites also stipulate illnesses be from one or more households. The average percentage of outbreak-associated infections during 2016–2018 were, by pathogen, *Campylobacter* (<1%), *Salmonella* (7%), STEC (4%), *Shigella* (5%), *Yersinia* (<1%), *Vibrio* (4%), *Listeria* (5%), *Cyclospora* (24%), and overall (4%).

^¶¶^ Cases per 100,000 persons.

*** Crude incidence is unadjusted and is calculated as cases of infection per 100,000 persons.

^†††^ A Bayesian, negative binomial model with penalized thin plate splines adjusting for state-specific trends was used to quantify adjusted incidence in 2023 and the IRR in 2023 compared with average incidence during 2016–2018. Incidence during 2023 was described as increased or decreased compared with 2016–2018 if the 95% CrI for the IRR did not include 1. A 95% CrI is analogous to a 95% CI in frequentist statistics and can be interpreted similarly, meaning a 95% probability of the true IRR for incidence in 2023 compared with average annual incidence during 2016–2018 is within the 95% CrI. https://www.medrxiv.org/content/10.1101/2022.09.14.22279742v1.full.pdf

**^§§§^** HP2030 incidence targets are based on domestically acquired infection incidence only. If the ill person did not report international travel or had unknown travel history, the illness was considered domestically acquired. A history of international travel was defined as travel ≤30 days before listeriosis and *Salmonella* Typhi and *S*. Paratyphi infection onset, ≤14 days before cyclosporiasis onset, and ≤7 days before onset for other infections. According to the Bayesian splines model, of the four illnesses with an HP2030 goal (*Campylobacter*, *Salmonella*, STEC, and *Listeria* infection), no evidence of a decrease was observed; instead, incidence of *Campylobacter* and STEC infection appears to have increased.

^¶¶¶^ Includes both international travel–associated infections and domestically acquired infections.

**** Infections that were not serotyped include all cases that were diagnosed by CIDT only, CIDT-diagnosed cases that failed to yield an isolate during reflex culture, and cases that yielded an isolate (both culture and CIDT-diagnosed) where the isolate was partially serotyped or not serotyped.

**^††††^** Dashes indicate that the given data point was unknown, not reported, otherwise missing from the FoodNet data, or was not quantified.

^§§§§^ Incidences for STEC O157 and overall STEC non-O157 represent only a proportion of the total STEC incidence because 1,425 (43%) infections yielded an isolate, and only 1,410 (42%) were fully serogrouped and classified as STEC O157 or STEC non-O157 during 2023. Thus, IRRs for STEC O157 and STEC non-O157 partially reflect the increasing proportion of STEC infections with unknown serogroup. Infections that were not serogrouped include all cases that were diagnosed by CIDT only, CIDT-diagnosed cases that failed to yield an isolate during reflex culture, and cases that yielded an isolate in which the isolate was partially serogrouped or not serogrouped.

**^¶¶¶¶^** The most frequently detected non-O157 serogroups were O103 (188) and O26 (173). The incidence of STEC O103 infection remained stable in 2023 compared with the 2016–2018 baseline (IRR = 0.89; 95% CrI = 0.70–1.14), and the incidence of STEC O26 infection decreased substantially (IRR = 0.75; 95% CrI = 0.63–0.90) during 2023.

***** Includes only domestically acquired infections (those for which the patient had no history of international travel or unknown travel history).

^†††††^ The incidence of domestically acquired STEC O103 infection remained stable in 2023 compared with the 2016–2018 baseline (IRR = 0.86; 95% CrI = 0.67–1.11), and the incidence of domestically acquired STEC O26 infection decreased substantially (IRR = 0.77; 95% CrI = 0.66–0.93) during 2023.

^§§§§§^ For ease of comparison with the HP2030 goal, the reported incidence of domestically acquired *Listeria* infections during 2023 is shown to the second decimal place.

Generally, the overall percentage of infections attributable to specific *Campylobacter, Shigella, Vibrio,* and *Yersinia* species, *Salmonella* serotypes, and STEC serogroups was lower in 2023 than in all previous years (Table 3). The overall incidence of infections for which the pathogen was not speciated, serotyped, or serogrouped increased substantially compared with incidence during 2016–2018 (Table 2). During 2023, 78% of all bacterial infections were diagnosed by CIDTs in the historic catchment area, including 46% diagnosed using only CIDTs. The percentage of CIDT-diagnosed infections for which a reflex culture*** was attempted decreased from 71% during 2016–2018 to 68% during 2023. This decrease was largest for *Yersinia*, *Vibrio*, and STEC infections. For all illnesses except listeriosis*,* the percentage of reflex cultures that yielded an isolate (successful [or positive] reflex culture) was lower in 2023 than during previous years (Table 3). This decrease in isolate availability has been associated with a decrease in serotyped, serogrouped, and speciated infections. For example, from 2016–2018 to 2023, the overall incidence of unspeciated infections increased substantially for *Campylobacter*,^†††^ *Shigella*,^§§§^ *Yersinia*,^¶¶¶^ and Vibrio****; the percentage of speciated infections declined from 33% to 26% for *Campylobacter*, from 65% to 41% for *Shigella*, from 49% to 23% for *Yersinia*, and from 61% to 34% for *Vibrio*. Although only culture-independent methods are used to diagnose cyclosporiasis, increases in CIDT-diagnosed cyclosporiasis and cyclosporiasis incidence mirror CIDT-driven increases in bacterial infection incidence.

**TABLE 3:** Percentage of bacterial infections diagnosed only by culture-based methods, and by culture-independent diagnostic tests* in the historic^†^ catchment area during 2010–2015, 2016–2018, and 2023^§^^,^^¶^ — Foodborne Diseases Active Surveillance Network, United States, 2010–2018 and 2023

Pathogen	Total, no. (% of total)^††^			Diagnosis method, no. (% of species/serotype/serogroup total**)								
				CIDT						Culture-based methods only (Cx+)		
				Any CIDT^§§^			Positive reflex culture (CIDT+/Cx+)					
	2010–2015	2016–2018	2023	2010–2015	2016–2018	2023	2010–2015	2016–2018	2023	2010–2015	2016–2018	2023
***Campylobacter* ^¶¶^**												
All***	**44,698**	**27,977**	**11,926**	**6,517 (14.6)**	**14,867 (53.1)**	**9,704 (81.4)**	**1,157 (2.6)**	**4,912 (17.6)**	**2,867 (24.0)**	**38,181 (85.4)**	**13,110 (46.9)**	**2,222 (18.6)**
*C. jejuni*	**14,675 (32.8)**	**8,024 (28.7)**	**2,672 (22.4)**	**—^†††^**	—^†††^	—^†††^	908 (6.2)	3,397 (42.3)	2,155 (80.7)	13,767 (93.8)	4,627 (57.7)	517 (19.3)
*C. coli*	**1,399 (3.1)**	**841 (3.0)**	**316 (2.6)**	**—^†††^**	—^†††^	—^†††^	69 (4.9)	365 (43.4)	247 (78.2)	1,330 (95.1)	476 (56.6)	69 (21.8)
*C. upsaliensis*	**332 (0.7)**	**188 (0.7)**	**79 (0.7)**	**—^†††^**	—^†††^	—^†††^	10 (3.0)	115 (61.2)	74 (93.7)	332 (100.0)	73 (38.8)	5 (6.3)
*C. lari*	**101 (0.2)**	**65 (0.2)**	**13 (0.1)**	**—^†††^**	—^†††^	—^†††^	1 (1.0)	14 (21.5)	7 (53.8)	100 (99.0)	51 (78.5)	6 (46.2)
*C. fetus*	**36 (0.1)**	**47 (0.2)**	**9 (0.1)**	**—^†††^**	—^†††^	—^†††^	0 (—)^†††^	1 (2.1)	1 (11.1)	36 (100.0)	46 (97.9)	8 (88.9)
Other species	**49 (0.1)**	**77 (0.3)**	**38 (0.3)**	**—^†††^**	—^†††^	—^†††^	6 (12.2)	28 (36.4)	11 (28.9)	43 (87.8)	49 (63.6)	27 (71.1)
Not speciated	**28,106 (62.9)**	**18,735 (67.0)**	**8,799 (73.8)**	**—^†††^**	—^†††^	—^†††^	163 (0.6)	992 (5.3)	372 (4.2)	22,583 (80.4)	7,788 (41.6)	1,590 18.1)
***Salmonella* ^§§§^**												
**All** ^¶¶¶^	**47,131**	**25,291**	**8,454**	**1,516 (3.2)**	**7,516 (29.7)**	**5,022 (59.4)**	**778 (1.7)**	**5,177 (20.5)**	**3,496 (41.4)**	**45,615 (96.8)**	**17,775 (70.3)**	**3,432 (40.6)**
*S. *Enteritidis	**8,396 (17.8)**	**4,034 (16.0)**	**1,597 (18.9)**	**—^†††^**	—^†††^	—^†††^	103 (1.2)	988 (24.5)	901 (56.4)	8,293 (98.8)	3,046 (75.5)	696 (43.6)
*S. *Newport	**5,258 (11.2)**	**2,407 (9.5)**	**566 (6.7)**	**—^†††^**	—^†††^	—^†††^	84 (1.6)	528 (21.9)	306 (54.1)	5,174 (98.4)	1,879 (78.1)	260 (45.9)
*S. *Typhimurium	**5,445 (11.6)**	**2,235 (8.8)**	**541 (6.4)**	**—^†††^**	—^†††^	—^†††^	92 (1.7)	615 (27.5)	324 (59.9)	5,353 (98.3)	1,620 (72.5)	217 (40.1)
*S. *Javiana	**4,261 (9.0)**	**1,880 (7.4)**	**324 (3.8)**	**—^†††^**	—^†††^	—^†††^	50 (1.2)	358 (19.0)	153 (47.2)	4,211 (98.8)	1,522 (81.0)	171 (52.8)
*S. *I 4,[5],12:i:-	**2,227 (4.7)**	**1,329 (5.3)**	**279 (3.3)**	**—^†††^**	—^†††^	—^†††^	51 (2.3)	398 (29.9)	179 (64.2)	2,176 (97.7)	931 (70.1)	100 (35.8)
Other serotypes	**18,300 (38.8)**	**9,520 (37.6)**	**2,878 (34.0)**	**—^†††^**	—^†††^	—^†††^	309 (1.7)	2,110 (22.2)	1,414 (49.1)	17,991 (98.3)	7,410 (77.8)	1,464 (50.9)
Not serotyped	**3,244 (6.9)**	**3,886 (15.4)**	**2,269 (26.8)**	**—^†††^**	—^†††^	—^†††^	89 (2.7)	180 (4.6)	219 (9.7)	2,417 (74.5)	1,367 (35.2)	524 (23.1)
STEC****												
**All^††††^**	**7,824**	**7,953**	**3,351**	**5,821 (74.4)**	**7,919 (99.6)**	**3,348 (99.9)**	**4,577 (58.5)**	**4,493 (56.5)**	**1,422 (42.4)**	**2,003 (25.6)**	**34 (0.4)**	**3 (0.1)**
O157	**2,905 (37.1)**	**1,380 (17.4)**	**298 (8.9)**	**—^†††^**	—^†††^	—^†††^	1,932 (66.5)	1,357 (98.3)	295 (99.0)	973 (33.5)	23 (1.7)	3 (1.0)
Non-O157	**3,571 (45.6)**	**3,120 (39.2)**	**1,112 (33.2)**	**—^†††^**	—^†††^	—^†††^	2,605 (72.9)	3,109 (99.6)	1,112 (100.0)	966 (27.1)	11 (0.4)	0 (—)^†††^
Not serogrouped	**1,348 (17.2)**	**3,453 (43.4)**	**1,941 (57.9)**	**—^†††^**	—^†††^	—^†††^	40 (3.0)	27 (0.8)	15 (0.8)	64 (4.8)	0 (—)^†††^	0 (—)^†††^
***Shigella* ^§§§§^**												
**All^¶¶¶¶^**	**14,098**	**7,533**	**3,186**	**1,379 (9.8)**	**3,697 (49.1)**	**2,679 (84.1)**	**493 (3.5)**	**1,490 (19.8)**	**1,010 (31.7)**	**12,719 (90.2)**	**3,836 (50.9)**	**507 (15.9)**
*S. sonnei*	**10,093 (71.6)**	**3,411 (45.3)**	**364 (11.4)**	**—^†††^**	—^†††^	—^†††^	436 (4.3)	887 (26.0)	256 (70.3)	9,657 (95.7)	2,524 (74.0)	108 (29.7)
*S.* *flexneri*	**2,142 (15.2)**	**1,487 (19.7)**	**926 (29.1)**	**—^†††^**	—^†††^	—^†††^	41 (1.9)	525 (35.3)	644 (69.5)	2,101 (98.1)	962 (64.7)	282 (30.5)
*S. boydii*	**76 (0.5)**	**33 (0.4)**	**14 (0.4)**	**—^†††^**	—^†††^	—^†††^	2 (2.6)	10 (30.3)	8 (57.1)	74 (97.4)	23 (69.7)	6 (42.9)
*S. dysenteriae*	**24 (0.2)**	**5 (0.1)**	**3 (0.1)**	**—^†††^**	—^†††^	—^†††^	1 (4.2)	1 (20.0)	3 (100.0)	23 (95.8)	4 (80.0)	0 (—)^†††^
Not speciated	**1,326 (9.4)**	**2,597 (34.5)**	**1,879 (59.0)**	**—^†††^**	—^†††^	—^†††^	6 (0.5)	67 (2.6)	99 (5.3)	434 (32.7)	323 (12.4)	111 (5.9)
Yersinia*****												
**All^†††††^**	**951**	**1,293**	**1,437**	**26 (2.7)**	**898 (69.5)**	**1,325 (92.2)**	**4 (0.4)**	**297 (23.0)**	**247 (17.2)**	**925 (97.3)**	**395 (30.5)**	**112 (7.8)**
*Y. enterocolitica*	**771 (81.1)**	**554 (42.9)**	**294 (20.5)**	**—^†††^**	—^†††^	—^†††^	4 (0.5)	268 (48.4)	213 (72.4)	767 (99.5)	286 (51.6)	81 (27.6)
*Y. frederiksenii*	**39 (4.1)**	**30 (2.3)**	**15 (1.0)**	**—^†††^**	—^†††^	—^†††^	0 (—)^†††^	7 (23.3)	10 (66.7)	39 (100.0)	23 (77)	5 (33.3)
*Y.* *intermedia*	**17 (1.8)**	**33 (2.6)**	**8 (0.6)**	**—^†††^**	—^†††^	—^†††^	0 (—)^†††^	11 (33.3)	4 (50.0)	17 (100.0)	22 (66.7)	4 (50.0)
*Y.* *kristensenii*	**8 (0.8)**	**10 (0.8)**	**5 (0.4)**	**—^†††^**	—^†††^	—^†††^	0 (—)^†††^	1 (10.0)	2 (40.0)	8 (100.0)	9 (90)	3 (60.0)
*Y. pseudotuberculosis*	**10 (1.1)**	**6 (0.5)**	**2 (0.1)**	**—^†††^**	—^†††^	—^†††^	0 (—)^†††^	1 (16.7)	0 (—)^†††^	10 (100.0)	5 (83.3)	2 (100.0)
Other species	**41 (4.3**	**11 (0.9)**	**7 (0.5)**	**—^†††^**	—^†††^	—^†††^	0 (—)^†††^	3 (27.3)	4 (57.1)	41 (100,0)	8 (72.7)	3 (42.9)
Not speciated	**65 (6.8)**	**649 (50.2)**	**1,106 (77.0)**	**—^†††^**	—^†††^	—^†††^	0 (—)^†††^	6 (0.9)	14 (1.3)	43 (66.2)	42 (6.5)	14 (1.3)
***Vibrio* ^§§§§§^**												
**All^¶¶¶¶¶^**	**1,234**	**1,174**	**567**	**26 (2.1)**	**524 (44.6)**	**433 (76.4)**	**7 (0.6)**	**164 (14.0)**	**77 (13.6)**	**1,208 (97.9)**	**650 (55.4)**	**134 (23.6)**
*V. parahaemolyticus*	**692 (56.1)**	**403 (34.3)**	**105 (18.5)**	**—^†††^**	—^†††^	—^†††^	4 (0.6)	112 (27.8)	52 (49.5)	688 (99.4)	290 (72.0)	53 (50.5)
*V. alginolyticus*	**151 (12.2)**	**109 (9.3)**	**29 (5.1)**	**—^†††^**	—^†††^	—^†††^	0 (—)^†††^	1 (0.9)	0 (—)^†††^	151 (100.0)	108 (99.1)	29 (100.0)
*V. vulnificus*	**120 (9.7)**	**72 (6.1)**	**13 (2.3)**	**—^†††^**	—^†††^	—^†††^	0 (—)^†††^	1 (1.4)	0 (—)^†††^	120 (100.0)	71 (98.6)	13 (100.0)
*V. cholerae*	**68 (5.5)**	**73 (6.2)**	**20 (3.5)**	**—^†††^**	—^†††^	—^†††^	1 (1.5)	21 (28.8)	13 (65.0)	67 (98.5)	52 (71.2)	7 (35.0)
*V. fluvialis*	**73 (5.9)**	**55 (4.7)**	**25 (4.4)**	**—^†††^**	—^†††^	—^†††^	1 (1.4)	8 (14.5)	8 (32.0)	72 (98.6)	47 (85.5)	17 (68.0)
Other species	**84 (6.8)**	**25 (2.1)**	**5 (0.9)**	**—^†††^**	—^†††^	—^†††^	1 (1.2)	5 (20)	1 (20.0)	83 (98.8)	20 (80.0)	4 (80.0)
Not speciated	**692 (56.1)**	**437 (37.2)**	**370 (65.3)**	**—^†††^**	—^†††^	—^†††^	0 (—)^†††^	16 (3.7)	3 (0.8)	27 (58.7)	62 (14.2)	11 (3.0)
Listeria monocytogenes******	**738**	**420**	**163**	**4 (0.5)**	17 (4.0)	44 (27.0)	4 (0.5)	15 (3.6)	42 (25.8)	734 (99.5)	403 (96.0)	119 (73.0)
**Total**	**116,674**	**71,641**	**29,084**	**15,289 (13.1)**	**35,438 (49.5)**	**22,555 (77.6)**	**7,020 (6.0)**	**16,548 (23.1)**	**9,161 (31.5)**	**101,385 (86.9)**	**36,203 (50.5)**	**6,529 (22.4)**

**Abbreviations:** CIDT = culture-independent diagnostic test; CIDT+ = positive CIDT result; Cx = culture-based method; Cx+ = positive culture result; Cx– = negative culture result; FoodNet = Foodborne Diseases Active Surveillance Network; HP2030 = Healthy People 2030; STEC = Shiga toxin-producing *Escherichia coli*.

* Bacterial infections could be diagnosed by culture-based methods only (Cx+), by CIDTs only (CIDT+), or by CIDTs with a reflex culture. When diagnosed by CIDTs with a reflex culture, the culture could either yield an isolate (CIDT+/Cx+) or fail to yield an isolate (CIDT+/Cx–). Because speciation and subtyping are only possible when an isolate is available, species and subtype data are only reported in the table for infections diagnosed by culture and by CIDTs with a reflex culture that yielded an isolate (CIDT+/Cx+). To facilitate readability, data for cases diagnosed by CIDTs only (i.e., where no reflex culture was performed) is not shown; these data can be calculated by subtracting the values in the footnotes for negative reflex culture (CIDT+/Cx–) and the Positive Reflex Culture (CIDT+/Cx+) column from the Any CIDT column.

^†^ The historic catchment area includes those sites under surveillance since 2004, including Connecticut, Georgia, Maryland, Minnesota, New Mexico, Oregon, Tennessee, and selected counties in California (three), Colorado (seven), and New York (34).

^§^ Periods were selected to facilitate comparison of 2023 data with 1) 2016–2018, the reference period used by HP2030, and 2) 2010–2015, when CIDTs were not widely available or used. Comparing 2023 data with these two periods shows increased CIDT availability and adoption have affected the reported frequency of different bacterial pathogens in the FoodNet catchment area.

^¶^ Case data for 2023 are preliminary.

** Percentage of cases linked to a specific species or subtype that were diagnosed by the given method. For example, in 2023, 81% of *C. jejuni* cases were diagnosed by CIDTs followed by reflex culture, and 19% were diagnosed using culture-based methods only.

^††^ Total number of isolates reported during that time. To obtain the average annual number reported, divide this total by the number of years. For example, 22% of *Campylobacter* isolates in 2023 were identified as *C. jejuni *(2,672), whereas 29% were identified as *C. jejuni* during 2016–2018 (8,024, or an average of 2,675 per year).

^§§^ Includes cases diagnosed by CIDTs only (CIDT+) or by CIDTs with either a positive (CIDT+/Cx+) or negative (CIDT+/Cx–) reflex culture.

^¶¶^ During 2010–2023, the other *Campylobacter* species reported, in order of frequency, were *C. ureolyticus, C. concisus, C. hyointestinalis, C. curvus, C. gracilis, C. rectus, C. sputorum, C. helveticus, C. lanienae, C. mucosalis, C. showae, C. hominis, C. paraureolyticus*, and *C. pelordis*.

*** During 2010–2015, 5% (2,404) of reported *Campylobacter* infections were diagnosed by CIDT and had a negative reflex culture (CIDT+/Cx–). During 2016–2018, 14% (3,947) of reported *Campylobacter* infections were diagnosed by CIDT+/Cx–. During 2023, 21% (2,484) of reported *Campylobacter* infections were diagnosed by CIDT+/Cx–.

**^†††^** Dashes indicate that the given data point could not be quantified because of the absence of isolates for cases diagnosed by CIDT in which reflex culture was not performed or reflex culture was negative. Illnesses diagnosed by CIDT in which reflex culture was not performed or reflex culture was negative lack an isolate. As a result, these infections cannot be attributed to a specific species, serotype or serogroup.

^§§§^ During 2010–2023, other commonly identified *Salmonella* serotypes (at least 1,000 cases) were, in order of frequency: Infantis, Saintpaul, Muenchen, Montevideo, Braenderup, Oranienburg, Thompson, Heidelburg, Bareilly, Mississippi, and I 13,23:b:-.

^¶¶¶^ During 2010–2015, 0.3% (155) of reported *Salmonella* infections were diagnosed by CIDT+/Cx–. During 2016–2018, 3% (730) of reported *Salmonella* infections were diagnosed by CIDT+/Cx–. During 2023, 8% (707) of reported *Salmonella* infections were diagnosed by CIDT+/Cx–.

**** During 2010–2023, the most common non-O157 STEC subtypes reported (more than 500 isolates), in order of frequency, were O26, O103, O111, and O121.

**^††††^** During 2010–2015, 11% (860) of reported STEC infections were diagnosed by CIDT+/Cx–. During 2016–2018, 31% (2,442) of reported STEC infections were diagnosed by CIDT+/Cx–. During 2023, 33% (1,094) of reported STEC infections were diagnosed by CIDT+/Cx–.

^§§§§^ The increase in reflex cultures that fail to yield an isolate might be partially attributable to the fact that existing CIDT panels cannot distinguish between *Shigella* and enteroinvasive *E. coli*.

^¶¶¶¶^ During 2010–2015, 1% (204) of reported *Shigella* infections were diagnosed by CIDT+/Cx–. During 2016–2018, 14% (1,073) of reported *Shigella* infections were diagnosed by CIDT+/Cx–. During 2023, 36% (1,151) of reported *Shigella* infections were diagnosed by CIDT+/Cx–.

***** During 2010–2023, the other *Yersinia* species reported, in order of frequency, were *Y. ruckeri, Y. massiliensis, Y. aldovae, Y. mollaretii, Y. aleksiciae, Y. rohdei*, and *Y. bercovieri*.

**^†††††^** During 2010–2015, 0.5% (5) of reported *Yersinia* infections were diagnosed by CIDT+/Cx–. During 2016–2018, 25% (322) of reported *Yersinia* infections were diagnosed by CIDT+/Cx–. During 2023, 31% (443) of reported *Yersinia* infections were diagnosed by CIDT+/Cx–.

^§§§§§^ During 2010–2023, the other *Vibrio* species reported, in order of frequency, were *V. mimicus, V. hollisae, V. furnissii, V. damsela, V. metschnikovii, V. cincinnatiensis, V. harveyi, V. navarrensis, V. fischeri*, and *V. metoecus*.

^¶¶¶¶¶^ During 2010–2015, 1% (12) of reported *Vibrio* infections were diagnosed by CIDT+/Cx–. During 2016–2018, 23% (272) of reported *Vibrio* infections were diagnosed by CIDT+/Cx–. During 2023, 37% (211) of reported *Vibrio* infections were diagnosed by CIDT+/Cx–.

****** During 2010–2015, no *Listeria monocytogenes* infections were diagnosed by CIDT+/Cx–. During 2016–2018, 0.5% (2) of reported *L. monocytogenes* infections were diagnosed by CIDT+/Cx–. During 2023, 0.6% (1) of reported *L. monocytogenes* infections were diagnosed by CIDT+/Cx–.

### *Salmonella *Infections

Of 8,454 total (i.e., both domestically acquired and travel-associated) *Salmonella* infections during 2023 in the historic catchment area, 83% yielded an isolate; 89% of isolates were fully serotyped. The incidence of nonserotyped infections increased substantially.^††††^ The incidences of the most frequently reported serotypes, *S. *Enteritidis and *S. *Newport, remained stable during 2023 compared with those during 2016–2018, whereas the incidences of the next-most frequently reported serotypes, *S. *Typhimurium, *S. *Javiana, and *S. *I 4,[5],12:i:- decreased substantially.

### STEC Infections

Of 3,351 total STEC infections in the historic catchment area during 2023, 57% yielded an isolate; 87% of isolates were fully serogrouped. The incidence of nonserogrouped infections increased substantially in 2023 compared with that during 2016–2018.^§§§§^ During 2023, STEC O157 incidence decreased compared with incidence during 2016–2018, and non-O157 STEC incidence remained stable.

### Hemolytic Uremic Syndrome

During 2022, FoodNet identified 61 cases of postdiarrheal HUS in persons aged <18 years, including 39 among children aged <5 years. The incidence of postdiarrheal HUS among persons aged <18 years (0.6 per 100,000 persons) and those aged <5 years (1.4 per 100,000) remained stable in 2022 compared with that during 2016–2018.^¶¶¶¶^

## Discussion

The current findings and previous FoodNet reports (*2*,*3*) suggest a lack of progress toward foodborne disease reduction goals; however, this outcome might reflect changing diagnostic practices such as the increased use of CIDTs rather than an actual increase in disease incidence. Increased use of CIDTs facilitates prompt clinical diagnosis and treatment but also complicates the interpretation of surveillance data and trends because CIDT adoption has varied over time, among clinical labs, and by pathogen. In addition, although CIDTs are generally considered more sensitive than are culture-based methods, some have high false-positive rates for certain pathogens (e.g., *Vibrio*) (*4*–*6*). Previous studies have indicated that increased CIDT use has resulted in the diagnosis of infections that previously would have gone undetected; increased use of CIDTs has been associated with marked increases in reported incidence (*4*,*7*).

Increases in CIDT-diagnosed infections are also associated with decreased rates of reflex culture, thereby reducing the number of isolates available for subtyping, whole genome sequencing, and antimicrobial resistance characterization (*8*). The impact of this reduction differs by species, serotype, and serogroup. Because an isolate is required for speciation, serotyping, and serogrouping, reduced isolate availability might result in underdetection of illnesses attributable to specific *Campylobacter, Shigella, Vibrio,* and *Yersinia* species, *Salmonella* serotypes, and STEC serogroups. The substantial increase in the incidence of infections for which the pathogen was not speciated, serotyped, or serogrouped is likely an artifact of changing diagnostic practices (i.e., increased CIDT use), resulting in a reduced availability of isolates for speciation and typing. Continued reductions in isolate availability might hinder outbreak identification and response (e.g., whole genome sequencing–based cluster identification and source attribution), detection of emerging antimicrobial resistance, and tracking of trends in illnesses attributable to specific species, subtypes, serotypes, and resistant strains. Increasing successful reflex culture rates after a CIDT diagnosis is a public health priority, which requires focused efforts and resources at the federal, state, and local levels. 

FoodNet data are used to track trends in enteric illness, monitor progress toward disease reduction goals, and guide food safety policy*****^,^^†††††^ (*1*). Because FoodNet is a sentinel surveillance system representing 10 sites, national extrapolation relies on strong assumptions of representativeness. Although the sites included in the FoodNet catchment area were selected nonrandomly, past analyses suggest that FoodNet’s catchment area is broadly representative of the national population (*9*,*10*). Previously, the only notable difference between FoodNet’s historic catchment area and the national population identified by these studies was that Hispanic persons were underrepresented in the catchment area relative to national representation (*9*,*10*). Investigating enteric disease epidemiology for AI/AN and NH/PI persons using FoodNet data has also been complicated by the small size of these populations in the historic catchment area. By increasing representation for these specific populations in the FoodNet catchment area, FoodNet’s expansion has helped to partially alleviate these limitations and improve the generalizability of FoodNet data. Additional expansion might be needed as national and catchment area demographics change.

### Limitations

The findings in this report are subject to at least three limitations. First, underreporting might affect case counts because ill persons must seek care and be tested for their illness to be recorded as a case. Second, although ill persons might meet the FoodNet criteria for hospitalization or death, the underlying reason for hospitalization or death might be unknown. Deaths that occurred >1 week after specimen collection among nonhospitalized persons or after discharge for hospitalized persons might not be recorded. Finally, domestically acquired cases might be overestimated because of the inclusion of persons with unknown travel status.

### Implications for Public Health Practice

FoodNet’s surveillance efforts are critical for tracking foodborne and enteric illnesses in the United States. During 2023, FoodNet expanded its catchment area for the first time since 2004, and it now includes all of Colorado. This expansion improved the representativeness of the FoodNet catchment area, and the ability of FoodNet to monitor trends in disease incidence, including the impact of changing diagnostic practices and the generalizability of FoodNet data. Continued surveillance is needed to monitor the impact of changing diagnostic practices on disease trends and evaluate the efficacy of prevention efforts in reducing incidence.

## Footnotes

* FoodNet is a collaboration among CDC, 10 state health departments, the U.S. Department of Agriculture’s Food Safety and Inspection Service, and the Food and Drug Administration. The historic catchment area includes sites under surveillance since 2004, including Connecticut, Georgia, Maryland, Minnesota, New Mexico, Oregon, and Tennessee, and counties in California (three), Colorado (seven), and New York (34). The expanded catchment area includes these sites and 57 Colorado counties not in the historic catchment area (i.e., the rest of Colorado).

† FoodNet reviews hospital discharge data for pediatric HUS cases to validate surveillance reports and identify additional cases using *International Classification of Diseases, Tenth Revision* and *International Classification of Diseases, Eleventh Revision* codes.

§ 45 C.F.R. part 46.102(l)(2), 21 C.F.R. part 56; 42 U.S.C. Sect. 241(d); 5 U.S.C. Sect. 552a; 44 U.S.C. Sect. 3501 et seq.

¶ Incidence (cases per 100,000 persons) was calculated by dividing number of infections during 2023 by 2022 U.S. Census Bureau population estimates. Changes in incidence for the historic catchment area were quantified as described previously (https://www.medrxiv.org/content/10.1101/2022.09.14.22279742v1). Because only 1 year of expanded catchment area data are available, incidence changes could not be quantified for the expanded catchment area.

** If the ill person did not report international travel or had an unknown travel history, the illness was considered to have been domestically acquired. A history of international travel was defined as travel ≤30 days before listeriosis and *S.* Typhi and *S.* Paratyphi infections onset, ≤14 days before cyclosporiasis onset, and ≤7 days before onset for other infections.

†† In contrast to frequentist statistics, which use significance testing, in a Bayesian model, true significance testing is not done, and differences are described as substantial.

§§ Unknown responses were included in proportion denominators.

¶¶ Incidence was similar in the historic and expanded catchment areas during 2023 with the exception of a higher cyclosporiasis incidence and a higher percentage of outbreak-associated cases in the expanded catchment area because of a cyclosporiasis outbreak that affected the newly enrolled Colorado counties. https://cdphe.colorado.gov/press-release/cdphe-investigating-cyclospora-outbreak-on-western-slope

*** CIDTs do not require culturing for diagnosis; however, reflex culture might be attempted after CIDT-based diagnosis. Reflex culture refers to attempting to grow and isolate the detected pathogen in a laboratory culture medium after a positive CIDT test result. Reflex culture practices vary by diagnostic laboratory, state, and pathogen.

††† The most frequently reported *Campylobacter* species in the historic catchment area during 2023 were *C. jejuni* (2,672), *C. coli* (316), *C. upsaliensis* (79), *C ureolyticus* (19), and *C. lari* (13). Compared with 2016–2018, incidence of unspeciated campylobacteriosis increased substantially in 2023 (IRR = 1.31; 95% CrI = 1.19–1.45).

§§§ The most frequently reported *Shigella* species in the historic catchment area during 2023 were *S. flexneri* (926), *S. sonnei* (364), *S. boydii* (14), and *S. dysenteriae* (three). Incidence of unspeciated shigellosis increased substantially in 2023 compared with 2016–2018 (IRR = 2.33; 95% CrI = 1.79, 3.12).

¶¶¶ The most frequently reported *Yersinia* species in the historic catchment area during 2023 were *Y. enterocolitica* (294), *Y. frederiksenii* (28), *Y. kristensenii* (19), *Y. intermedia* (eight), and *Y. massilkensis* (four). Compared with 2016–2018, incidence of unspeciated yersinosis increased substantially in 2023 (IRR = 7.19; 95% CrI = 4.78–11.97).

**** The most frequently reported *Vibrio* species in the historic catchment area during 2023 were *V. parahaemolytics* (105), *V. alginolytics* (30), *V. fluvialis* (25), *V. cholerae* (20), and *V. vulnificus* (13). Compared with 2016–2018, incidence of unspeciated vibriosis increased substantially in 2023 (IRR = 4.29; 95% CrI = 3.00–6.59).

†††† Overall, the most frequently reported *Salmonella* serotypes in the historic catchment area during 2023 were not serotyped infections (2,297), *S*. Enteritidis (1,597), *S*. Newport (566), *S*. Typhimurium (541), *S*. Javiana (324), and *S*. I 4,[5],12:i:- (279). The IRR for nonserotyped, domestically acquired infections in the historic catchment area was 1.67 (95% CrI = 1.38–2.01).

§§§§ STEC isolates were considered fully serogrouped if the O antigen was determined. Overall, infections in which the isolate was not serogrouped (1,941) were more frequent than infections with fully serogrouped isolates (1,410); among all fully serogrouped isolates, serogroup O157 (298) was most frequently reported in the historic catchment area during 2023, followed by O103 (188), O26 (173), and O111 (160). The IRR for nonserogrouped, domestically acquired infections during 2023 compared with 2016–2018 was 1.79 (95% CrI = 1.36–2.48).

¶¶¶¶ The IRRs for pediatric HUS cases during 2022 compared with 2016–2018 for persons aged <18 years and <5 years were 1.0 (95% CrI = 0.8–1.2) and 1.0 (95% CrI = 0.8–1.3), respectively.

*****
https://www.fsis.usda.gov/inspection/inspection-programs/inspection-poultry-products/reducing-salmonella-poultry

†††††
https://www.fsis.usda.gov/policy/federal-register-rulemaking/federal-register-notices/changes-salmonella-verification-0
